# Transcriptome analysis provides insights into the molecular mechanism of *GhSAMDC*_*1*_ involving in rapid vegetative growth and early flowering in tobacco

**DOI:** 10.1038/s41598-022-18064-4

**Published:** 2022-08-10

**Authors:** Xinqi Cheng, Fangqin Pang, Wengang Tian, Xinxin Tang, Lan Wu, Xiaoming Hu, Huaguo Zhu

**Affiliations:** 1 College of Biology and Agricultural Resources, Huanggang Normal University, Huanggang, 438000 Hubei China; 2 Hubei Key Laboratory of Economic Forest Germplasm Improvement and Resources Comprehensive Utilization, Huanggang, 438000 Hubei China; 3 College of Agronomy, Shihezi University, Shihezi, 832000 Xinjiang China

**Keywords:** Molecular biology, Plant sciences

## Abstract

In previous study, ectopic expression of *GhSAMDC*_*1*_ improved vegetative growth and early flowering in tobacco, which had been explained through changes of polyamine content, polyamines and flowering relate genes expression. To further disclose the transcript changes of ectopic expression of *GhSAMDC*_*1*_ in tobacco, the leaves from wild type and two transgenic lines at seedling (30 days old), bolting (60 days old) and flowering (90 days old) stages were performed for transcriptome analysis. Compared to wild type, a total of 938 differentially expressed genes (DEGs) were found to be up- or down-regulated in the two transgenic plants. GO and KEGG analysis revealed that tobacco of wild-type and transgenic lines were controlled by a complex gene network, which regulated multiple metabolic pathways. Phytohormone detection indicate *GhSAMDC*_*1*_ affect endogenous phytohormone content, ABA and JA content are remarkably increased in transgenic plants. Furthermore, transcript factor analysis indicated 18 transcript factor families, including stress response, development and flowering related transcript factor families, especially AP2-EREBP, WRKY, HSF and Tify are the most over-represented in those transcript factor families*.* In conclusion, transcriptome analysis provides insights into the molecular mechanism of *GhSAMDC*_*1*_ involving rapid vegetative growth and early flowering in tobacco.

## Introduction

Polyamines (PAs) are low molecular weight aliphatic nitrogenous bases with biological activity, which contain two or more amino groups^1^, and mainly include putrescine (Put), spermidine (Spd), spermine (Spm) and thermospermine (T-Spm) in plants^2,3^. PAs showed tissue and organ specific distribution patterns^4,5^, and played crucial roles in various physiological processes in plants, some reports had been showed PAs involved in abiotic/biotic stress response^6–12^, leaf development and senescence^13–15^, fruit development and ripening^16–18^ and floral initiation and development^19–22^. Beside, many reports have indicated PAs as signaling molecules or mediated through the products of their catabolism together with plant hormone^23^, such as jasmonic acid (JA)^24^, abscisic acid (ABA)^25,26^, salicylic acid (SA)^27^ and ethylene^28^ were involved in various physiological processes.

In plants, Put is synthesized through arginine decarboxylase (ADC) and/or ornithine decarboxylase (ODC), and conversion of Put to Spd and Spm requires Spd synthase and Spm synthase, respectively^7^. *S*-adenosylmethionine decarboxylase (SAMDC) involve in the biosynthesis of decarboxylated SAM, which donates the aminopropyl moiety for the biosynthesis of these PAs. On the other hand, catabolism of PAs involves diamine oxidases (CuAOs) and polyamine oxidases (PAOs). The homeostasis of cellular PAs levels, being well regulated by a dynamic balance of biosynthesis and catabolism, and it is important for maintaining normal growth and development in plants. The functions of *SAMDC* genes had been reported in many plants, which indicate it play important roles in stress tolerance and plant development through regulating in polyamine synthesis^2,10,13,22^.

In previous study, an upland cotton S-adenosylmethionine decarboxylase (*GhSAMDC*_*1*_, GenBank NO. JN020148) gene was transformed into tobacco and resulted in rapid vegetative growth and early flowering. To further disclose the potential molecular mechanism of *GhSAMDC*_*1*_, a transcriptome analysis was performed in wild type and transgenic plants at different stages.

## Results

### Phenotype identification of transgenic plants

**Figure 1:**
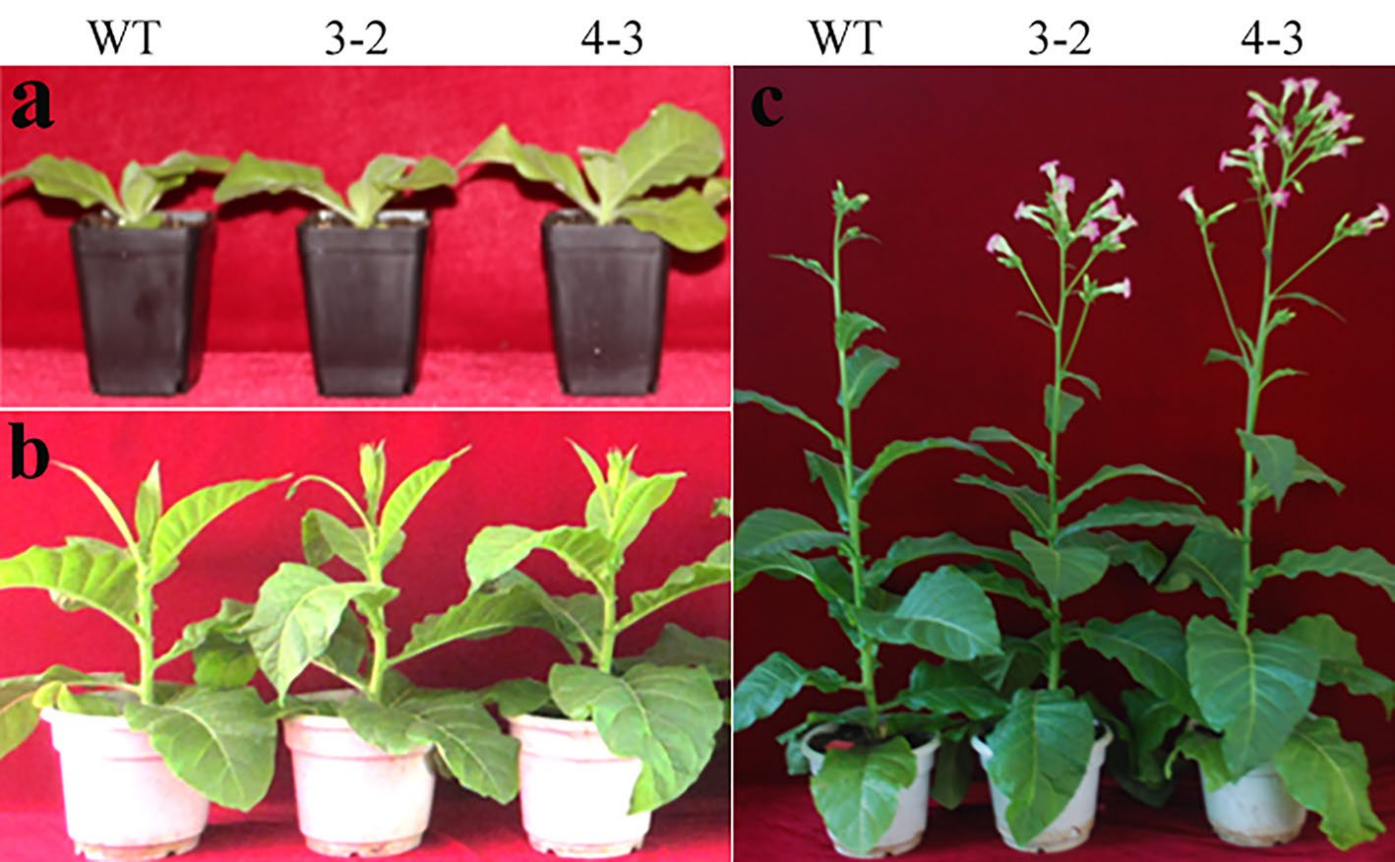
Phenotype of wild type and transgenic plants. (**a–c**) Phenotype of wild type and transgenic plants at seedling, bolting and flowering stages, respectively.

In previous study, transgenic plants (3–1, 3–2, and 4–3) from three independent transformation events had been analyzed and identified^22^. Compared to wild type, transgenic plants showed rapid vegetative growth in early bolting and flowering stage (Fig. 1A–C). Based on the previous expression analysis, we selected seedling stage (30 days, S) (Fig. 1A), bolting stage (60 days, B) (Fig. 1B) and flowering stage (90 days, F) (Fig. 1C) of wild type (WT) and transgenic (3–2 and 4–3) lines for transcription sequencing.

### Primary analysis of transcriptome data

**Figure 2:**
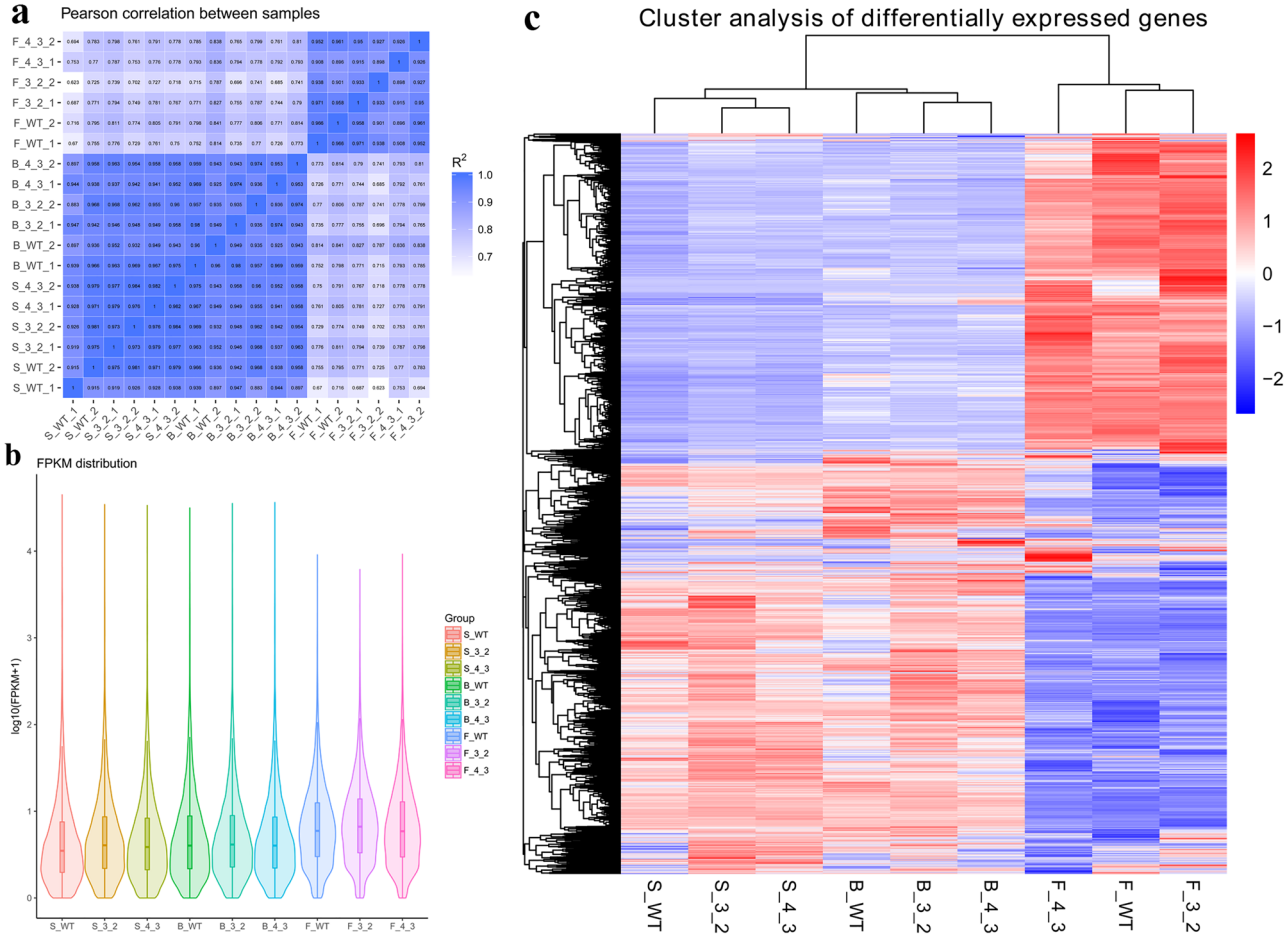
Primary analysis of transcriptome datas. (**a**) Heat map of Pearson correlation coefficient between samples; (**b**) Violin diagram of gene expression level in wild type and transgenic lines at different stages; (**c**) Heat map for cluster analysis of the differentially expressed transcripts. The color scale corresponds to the log_2_ (FPKM) values of genes in various samples, red represented up-regulated expression and blue represented down-regulated expression.

Transcriptome analysis indicated sequencing produced libraries averaging 57.83 million clean reads per library, and average of Q20 and Q30 value were 97.24% and 92.11%, respectively (Table S1). Heat map of Pearson correlation coefficient between different samples was shown in Fig. 2A, the coefficient were 0.915–0.982 between twice replicates, and details were described in supplementary. Principal Component Analysis (PCA) show that the clusters in different periods have large differences (Figure S1). Fragments Per Kilobase of transcript sequence per Millions base pairs sequenced (FPKM) distribution analysis suggested the percentage of FPKM interval 0–1, 1–3, 3–15, 15–60, > 60 are 54.52%, 14.96%, 21.76%, 6.69% and 2.08%. The violin diagram of gene expression level is shown in Fig. 2B, and details were described in supplementary Table S2. Based on the above data analysis, a cluster analysis of the differentially expressed transcripts was described in Fig. 2C.

### Gene expression analysis of WT and transgenic lines at different developmental stages

**Figure 3:**
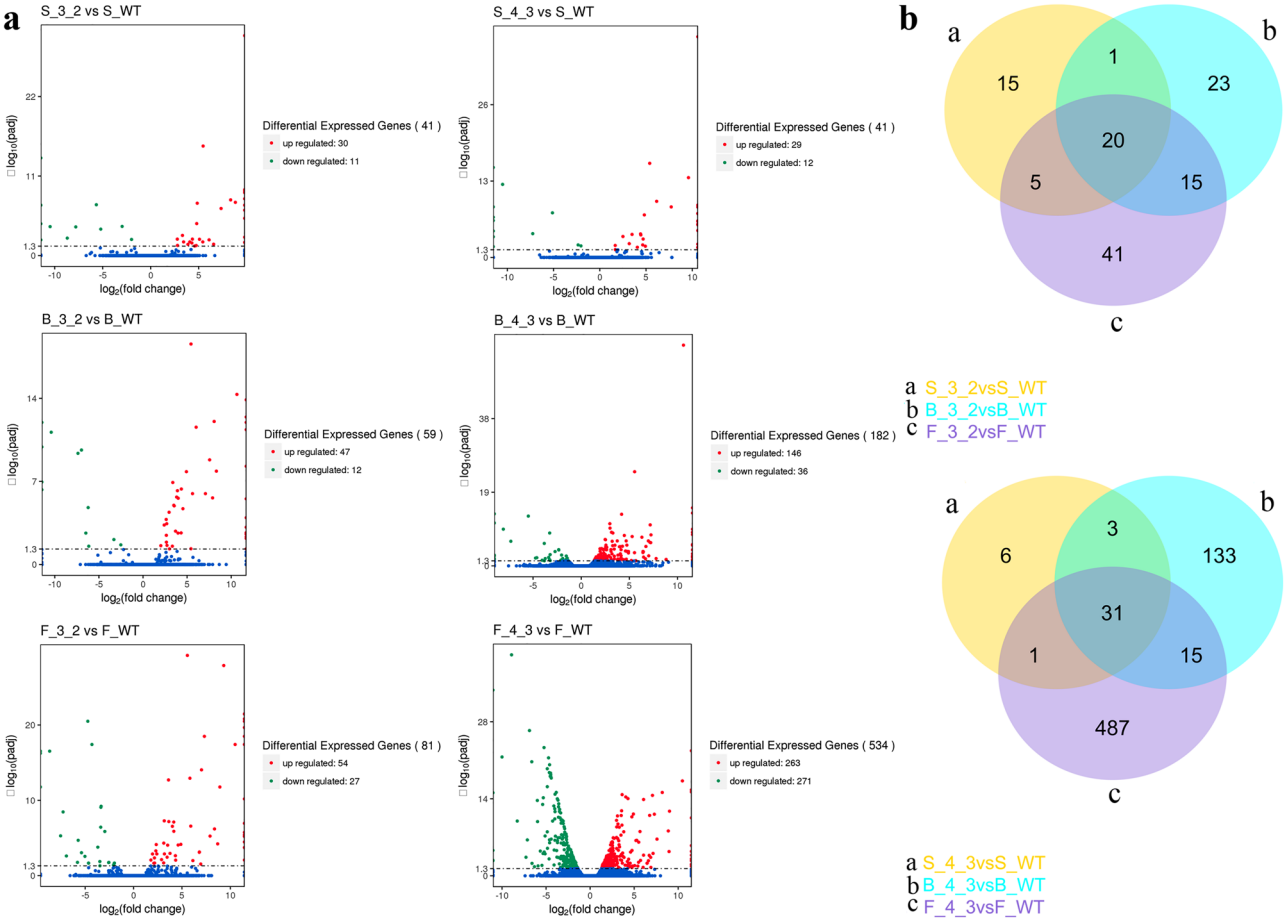
Analysis of gene expression differences between wild type and transgenic lines. (**a**) Diagram showing the number of genes up- and down-regulated genes between wild type and transgenic lines at different stages; (**b**) Venn diagrams for differentially expressed genes between wild type and transgenic lines.

Comparisons of gene expression between WT and the two transgenic lines were performed at different stages. Overall, there were 938 genes identified that were differentially expressed in the transgenic lines compared to the WT (|log _2_ (fold change) |≥ 2 and p_*adj*_ < 0.05). Among these genes, 82, 241 and 615 DEGs were acquired at seedling, bolting and flowering stages, respectively (Table S3). Compared to WT, transgenic line 3–2 had 181 DEGs (131 up- and 50 down- regulated), and transgenic line 4–3 had 757 DEGs (438 up- and 319 down-regulated), the details are described in Fig. 3A. As was shown in Fig. 3B, two three-way Venn diagrams of 3–2 *vs* WT and 4–3 *vs* WT at different stages were displayed, respectively. 120 were unique ones with 15, 23 and 41 unique to seedling, bolting and flowering stage in the transgenic line 3–2 and WT comparison, respectively. Of the 676 non-redundant DEGs in the transgenic line 4–3 compared to WT, there were 31 DEGs shared at different stages, there were 6, 133 and 487 genes to seedling, bolting and flowering stage in the transgenic line 4–3 and WT.

### GO and KEGG pathway analysis of DEGs between 4–3 and WT at different stages

**Figure 4:**
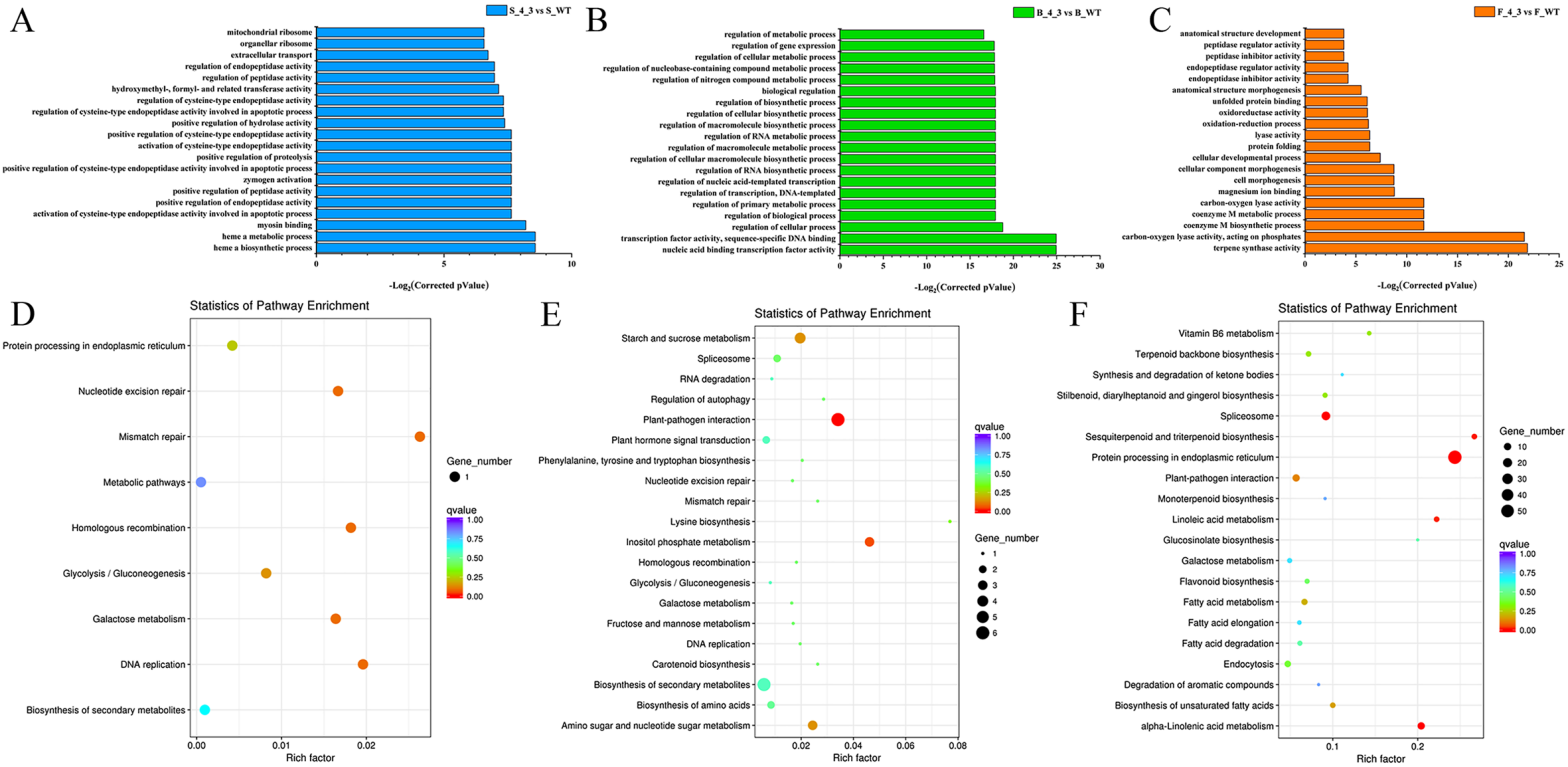
GO annotations and KEGG enrichmentanalysis of the DEGs between wild type and transgenic line 4–3 at different stages. (**a–c**) GO annotations of the DEGs between wild type and transgenic line 4–3 at different stages; (**d–f**) KEGG enrichment analysis of the DEGs between wild type and transgenic line 4–3 at different stages.

To gain a better understanding of the molecular mechanism of *GhSAMDC*_*1*_ involving in rapid vegetative growth and early flowering in tobacco, GO annotations of the DEGs and enrichment of KEGG pathway were performed in comparison 3–2 *vs* WT and 4–3 *vs* WT at different stages, it found that due to the small number of DEGs between 3 and 2 and WT, GO were not enriched at different stages. Therefore, we only compared between 4 and 3 and WT at different stages. GO annotations of the DEGs between WT plants and transgenic line 4–3 at different stages were presented in Fig. 4A–C, Using the criterion of corrected *P*_*Value*_ ≤ 0.05, a total of 49 GO terms were enriched at bolting stage, There were 41 GO terms involved in biological process level, mainly including regulation of cellular process, regulation of biological process, primary metabolic process, transcription, DNA-templated, nucleic acid-templated transcription, RNA biosynthetic process, cellular macromolecule biosynthetic process, macromolecule metabolic process, RNA metabolic process, nitrogen compound metabolic process, nucleobase-containing compound metabolic process. In cellular component level, DEGs were over-represented in proteinaceous extracellular matrix, extracellular matrix, extracellular region part, extracellular region. For molecular function level, over-represented DEGs were associated with nucleic acid binding transcription factor activity, sequence-specific DNA binding, inositol-3-phosphate synthase activity, intramolecular lyase activity (Fig. 4B, Table S4). 15 GO terms were enriched at flowering stage, 8 biological process terms were enriched were related to coenzyme M biosynthetic process, coenzyme M metabolic process, cell morphogenesis, cellular component morphogenesis, cellular developmental process, protein folding, oxidation–reduction process, anatomical structure morphogenesis, in molecular function level mainly involved terpene synthase activity, carbon–oxygen lyase activity, magnesium ion binding, lyase activity, oxidoreductase activity, unfolded protein binding. (Fig. 4C, Table S5). In order to further analyze the DEGs, we performed Kyoto Encyclopedia of Genes and Genomes (KEGG) pathways analysis (Fig. 4D-F), using the criterion of corrected *P *_*Value*_ ≤ 0.05, KEGG enrichment analysis indicated histidine metabolism between 3 and 2 and WT at bolting stage (Table S6). At the bolting stage in 4–3 *vs* WT, pathways related to plant-pathogen interaction (Fig. 4E, Table S6), at the flowering stage between 4 and 3 and WT, they were involved protein processing in endoplasmic reticulum, alpha-Linolenic acid metabolism, spliceosome, sesquiterpenoid and triterpenoid biosynthesis, linoleic acid metabolism (Fig. 4F, Table S6). These results indicate that tobacco of wild-type and transgenic are controlled by a complex gene network, which regulates multiple metabolic pathways.

### Protein–protein interaction (PPI) analysis

**Figure 5:**
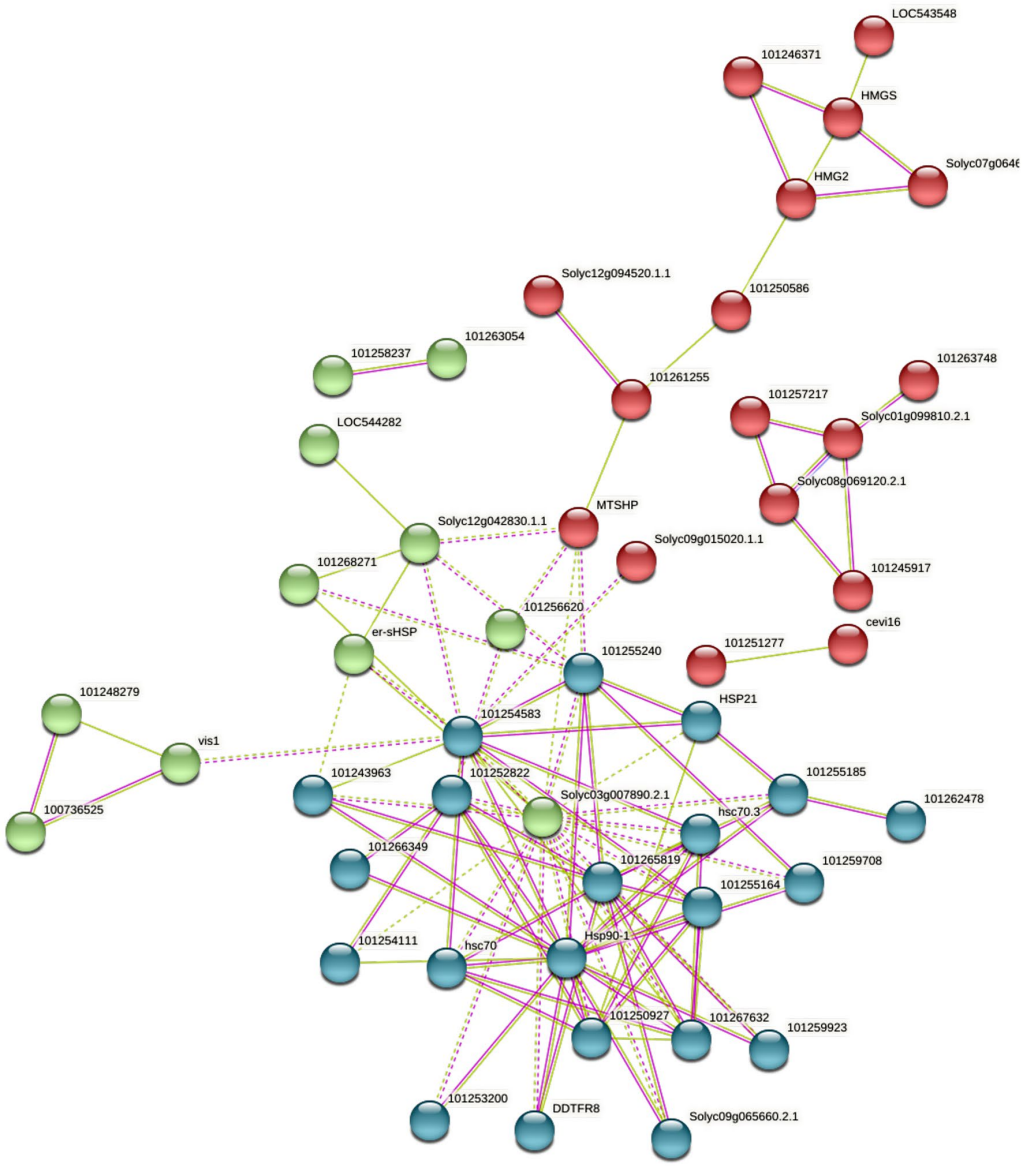
Protein–protein interaction network analysis of DEGs between transgenic 4–3 and wild type at flowering stage. The STRING database (http://string-db.org/) was used to analyze the protein–protein interaction network based on the proteins corresponding to all DEGs.

To further understand the relationship of DEGs, PPI network analyses of DEGs with FDR ≤ 0.05 and |Log_2_FC|≥ 2 was performed using STRING database. Through BlastX comparison, the interaction relationship among these DEGs was found and visualized in the String database. A total of 105 interaction relationships were identified among 77 DEGs between transgenic line 4–3 and WT at flowering stage (Fig. 5), which indicated average node degree was 2.73, average local clustering coefficient was 0.354, expected number of edges was 12 and PPI enrichment *P *_*value*_ was less than 1.0 × e^16^.The DEGs including Hsp90-1, hsc70 (Heat shock cognate 70 kDa protein 1), Solyc03g007890.2.1 (Heat shock protein 90), 101,252,822(Heat shock protein 70 family protein), 101,243,963 (Heat shock transcription factor B2A), 101,255,185 (Heat shock protein 70B), 101,267,632 (Winged-helix DNA-binding transcription factor family protein), 101,255,164 (Heat shock protein 70) and 101,265,819 (Chaperone protein htpG family protein) were found to play important roles in maintaining the tight connection of the whole network. The result indicated ectopic expression of *GhSAMDC*_*1*_ might involve in early flowering through activating heat shock protein in transgenic tobacco^29,30^.

### Quantitative RT-PCR validation of RNA-sequencing data

**Figure 6:**
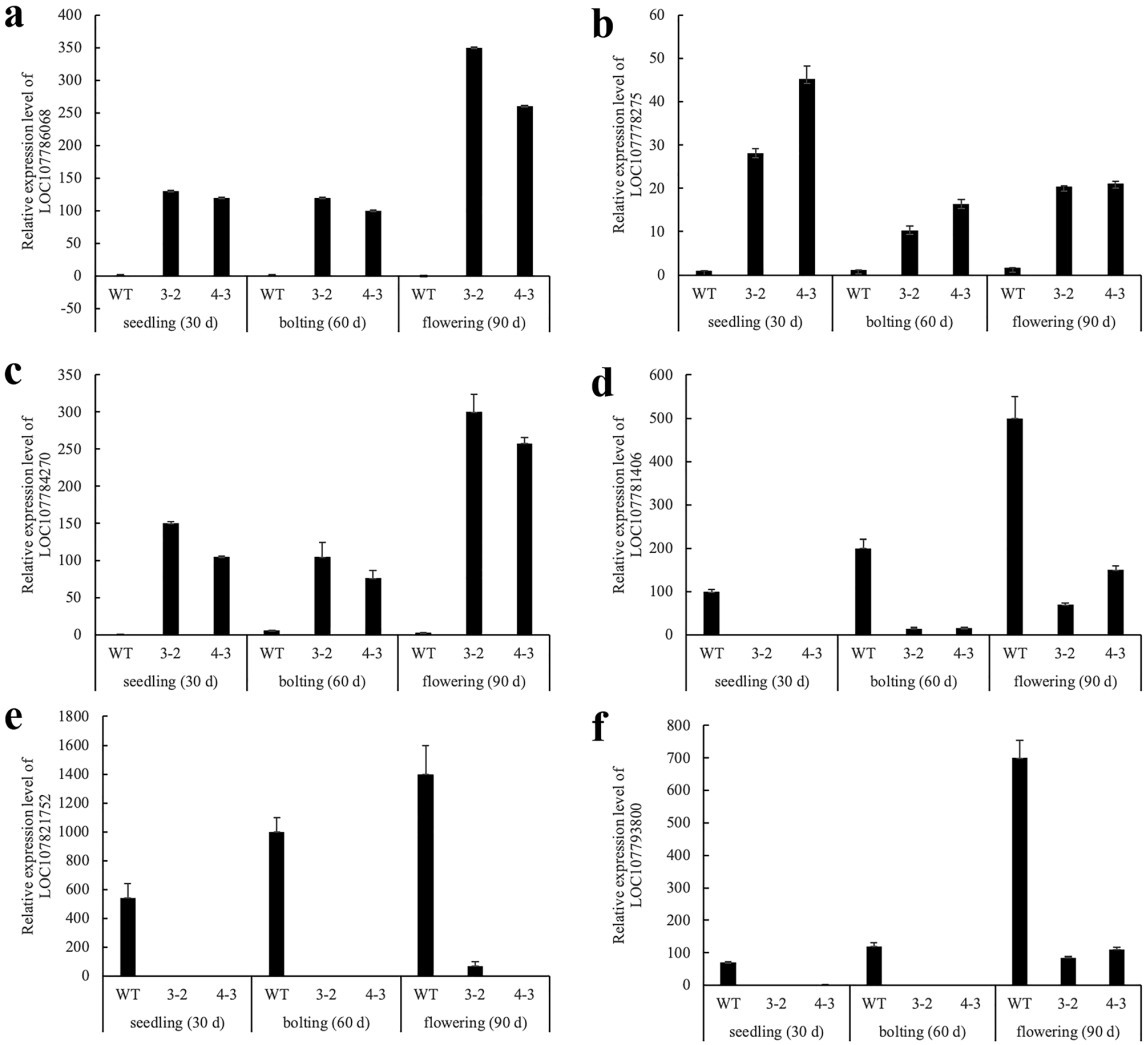
Relative expression analysis of 6 DEGs by qRT-PCR.

To confirm the accuracy of the transcriptome analysis results, real-time quantitative reverse transcription-PCR (qRT-PCR) was used to analyze DEGs identified in the RNA-Seq results. 6 DEGs between wild type and transgenic lines at different stages were selected for qRT-PCR analysis. There was a general agreement between the RNA-seq (Table S7) and qRT- PCR (Fig. 6) results for all genes analyzed validating the RNA-seq library.

## Discussion

### Interaction of polyamine and hormone affected plant growth and development

Based on our previous study, ectopic expression of *GhSAMDC*_*1*_ resulted in Spd content decrease and then increase accompany with Put decrease at flowering stage in transgenic lines, which indicates Spd plays an important role in floral induction^22^. Changes of PA content could alter gene expression level of genes associated with plant hormone signaling transduction in *Arabidopsi*s^31^, and Spd involved floral induction by decreasing GA3 and increasing MdNCED1 and MdNCED3 through ABA enrichment in apple^32^. Recently, plant CuAOs involving in polyamine terminal oxidation were induced by stress-related hormones, including methyl-jasmonate (MeJA), abscisic acid (ABA) and salicylic acid (SA), which indicated polyamine homeostasis was affected by plant hormone^33^. In thus, crosstalk between polyamine and hormone plays a crucial role in plant growth and development.

**Figure 7:**
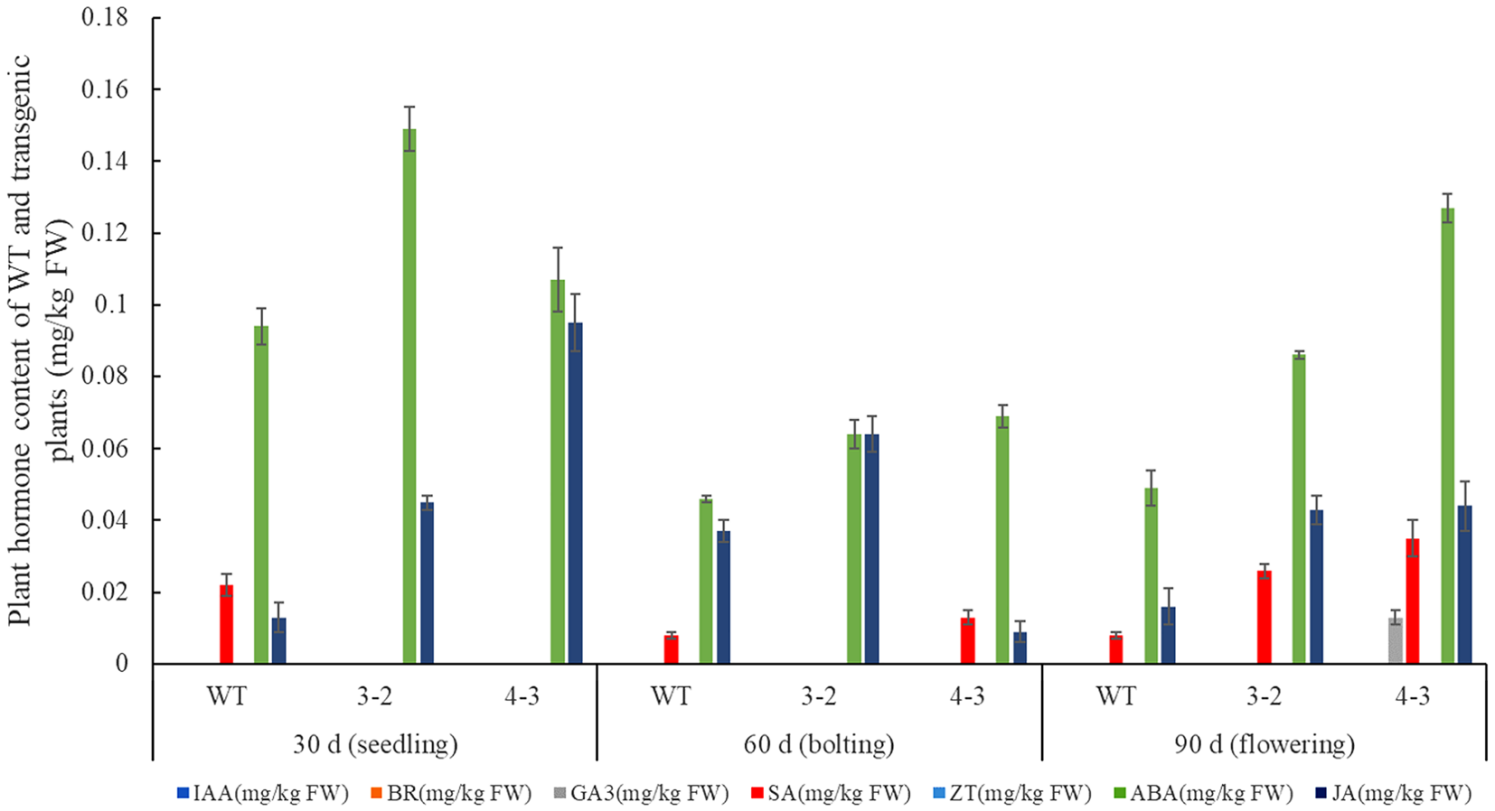
Plant hormone content of wild type and transgenic lines at different stages.

Phytohormone detection was performed in wild type and transgenic lines at different stages (Fig. 7). IAA, BR and ZT were not detected in all samples, GA3 was only detected in transgenic line 4–3 at flowering stage. GA are closely associated with DELLA involving in flowering pathways^34,35^, and a highly conserved family of R2R3 MYB transcription factors had been reported involving in the GA signaling pathway and flowering^36–38^. SA content was detected in all stages and decreased accompany with growth in wild type plants, whereas only was detected in later stages and showed reverse tendency in transgenic lines. Increase of Spd enhanced SA content in the leaves had been reported in wheat^39^, and vice versa SA treatment affects the PAs metabolism in plants^27,40–42^. In *Arabidopsis*, exogenous SA appears to be a repressor of the expression of flowering related gene FLC^43^. Remarkably, ABA and JA were detected in all samples, and the content of them in transgenic lines were higher than that in wild type plants, it was consistent with exogenous Spm induced JA accumulation in lima bean^24^. Furthermore, a positive feedback loop has also been found between ABA and PAs, ABA activates the PA metabolism as well as PA induces ABA synthesis^25,44,45^, and various miRNAs have also been documented to be involved in flowering by means of ABA signaling and regulation^46–48^. Further indicated a complicated relationship exist between PAs and plant hormones, and which play crucial role in ectopic expression of *GhSAMDC*_*1*_ involving rapid vegetative growth and early flowering in tobacco.

### Transcription factor genes affect rapid vegetative growth and early flowering in transgenic plants overexpressing *GhSAMDC*_*1*_

Analyzing GO annotation of the DEGs between 4 and 3 and WT at bolting stage found that there were enriched transcription factor activity, therefore, the transcription factor was analyzed. As is shown in Table S8, 18 transcription factor families were identified in those DEGs, and AP2-EREBP, WRKY, HSF and Tify were the most over represented families. In these families, AP2/EREBP including 14 genes was the most abundant transcription factor family. As we known, AP2/EREBP comprise AP2-like genes (with two AP2 domains) and EREBP-like or ERF-like genes (with one AP2 domain)^49^, and they are involved in plant development, especially reproductive growth through regulating ABA content^50,51^. In this study, compared to wild type, ABA content was remarkably increased in transgenic lines at different stages, which indicated increase of ABA content might be regulated by those AP2/EREBP genes. Furthermore, 9 WRKY transcription factor genes were identified and over-represented. Except for responding to biotic and abiotic stress^52,53^, WRKY genes are also involved in several physiological and developmental processes^54,55^. Especially, many studies have demonstrated that WRKY genes play crucial roles in flowering^56–59^. In addition, both HSF and Tify transcription factor family contained 6 genes, and they were also over-represented in those DEGs. HSF had been reported that they not only involve in abiotic stress response^60–62^, but also regulate plant development^63^ and flowering^29,30^. Tify are plant-specific transcription factors characterized by the presence of a highly conserved motif (TIF(F/Y)XG) in the TIFY domain^64^. Many studies had been reported they encodes proteins involve in multiple biological processes through modulating jasmonate signaling pathway^65^, including leaf development^66^, abiotic stress resistance^67–69^ and flowering^70,71^. Altogether, identification of abundant plant development and flowering related transcription factor genes indicated these genes at least partly participate regulating rapid vegetative growth and early flowering in ectopic expression of *GhSAMDC*_*1*_ in tobacco.

## Methods

### Plant materials and sampling

The tobacco variety used in this study was SR1 (*Nicotiana tabacum* pv SR1), and the plant was preserved and planted in the Hubei Key Laboratory of Economic Forest Germplasm Improvement and Resources Comprehensive Utilization. Wild type and homozygous T_3_ transgenic tobacco seeds (including 3–2 and 4–3) were surface sterilized with 2% NaClO and washed three times with sterile water. The sterile seeds were then suspended in 0.2% agar and plated on 1⁄2 Murashige and Skoog (MS) medium plus 1.5% sucrose^72^. 50 seedlings were selected from each variety and planted in artificial climate chamber (16 h light and 8 h dark cycle at 25 °C), observe the phenotypic changes of seedlings and leaves from the same position on 30 days (Seedling stage, S), 60 days (Bolting stage, B), 90 days (Flowering stage, F) and wild type (WT), transgenic lines 3–2 (3–2) and 4–3 (4–3) plants were sampled for transcriptome analysis (Table S9). Twice biological replicates were performed.

### Library preparation and Transcriptome sequencing

Total RNA was extracted using Trizol reagent following the manufacturer’s protocol. RNA purity was checked using the NanoPhotometer^®^ spectrophotometer (IMPLEN). RNA concentration was measured using Qubit^®^ RNA Assay Kit in Qubit^®^ 2.0 Flurometer. RNA integrity was assessed using the RNA Nano 6000 Assay Kit with the Bioanalyzer 2100 system. A total amount of 1 μg RNA per sample was used as input material for the RNA sample preparations. Sequencing libraries were generated using NEBNext^®^ UltraTM RNA Library Prep Kit for Illumina^®^ (NEB, USA) following manufacturer’s recommendations. The clustering of the index-coded samples was performed on a cBot Cluster Generation System using TruSeq PE Cluster Kit v3-cBot-HS (Illumia) according to the manufacturer’s instructions. After cluster generation, the library preparations were sequenced on an Illumina Hiseq platform and 125 bp/150 bp paired-end reads were generated.

### Data analysis

The datasets used and analysed have been deposited in the National Center for Biotechnology Information (NCBI). The accession number is PRJNA759726 (http://www.ncbi.nlm.nih.gov/bioproject/759726), which includes 18 accession items (SRR15709784—SRR15709801). Raw data of fastq format were firstly processed through in-house perl scripts. *Nicotiana tabacum* reference genome and gene model annotation files were downloaded from genome website directly (https://www.ncbi.nlm.nih.gov/genome/425?genome_assembly_id=274804). HTSeq v0.9.1 was used to count the reads numbers mapped to each gene. And then FPKM of each gene was calculated based on the length of the gene and reads count mapped to this gene, The DESeq software was used to determine differential gene expression by negative binomial distribution and calculation of false discovery rate (FDR) by the Benjamini and Hochberg method, as well as the adjusted *P*_*value*_. The adjusted *P*_*value*_ < 0.05 was used as the threshold for identification of DEGs among samples. Differential expression analysis of two conditions/groups (two biological replicates per condition) was performed using the DESeq R package (1.18.0) (https://bioconductor.org/packages/release/bioc/html/DESeq2.html). Genes with an adjusted *P*_*value*_ < 0.05 found by DESeq were assigned as differentially expressed. Gene Ontology (GO) enrichment analysis of differentially expressed genes was implemented by the clusterProfler R package (http://www.bioconductor.org/packages/release/bioc/html/clusterProfiler.html). GO terms with corrected *P*_*value*_ less than 0.05 were considered significantly enriched by differential expressed genes. KOBAS software was used to test the statistical enrichment of differential expression genes in KEGG pathways (www.kegg.jp/kegg/kegg1.html). PPI analysis of differentially expressed genes was based on the STRING database (https://cn.string-db.org/), which known and predicted Protein–Protein Interactions^73^, Nodes represent the DEGs enriched in the STRING database, while edges reflect the interactions between differentially expressed genes, the interaction data was imported into Cytoscape software v3.2.0 (http://cytoscape.org) to realize the visualization of the interaction network. Transcript factors analysis was performed using iTAK software online (http://itak.feilab.net/cgi-bin/itak/index.cgi).

### RNA isolation and quantitative reverse transcriptase-polymerase chain reaction (qRT-PCR) analysis

Total RNA was extracted from the samples using the modified CTAB method^22^. qRT-PCR was performed using Power SYBR Green Master (Roche, Basel, Switzerland) on a Roche Light Cycler 480 system (Roche), as described previously. The reaction was run as follows: pre-incubation at 94 °C for 2 min, 40 cycles of 94 °C for 20 s, 58 °C for 20 s and 72 °C for 20 s. The actin gene (Tac9; X69885) was used as the reference gene, and the relative 2^−∆ct^ quantification method was used to evaluate quantitative variation. Three biological replicates and three technical repeats were run. The qRT-PCR primers are listed in Table S10.

### Phytohormone detection

Leaves from wild type and transgenic lines 3–2 and 4–3 were sampled for phytohormone detection at seedling (30 days old), bolting (60 days old) and flowering (90 days old) stages, Three biological replicates were performed for each sample. Samples were rapidly frozen by liquid nitrogen and transported with dry ice for analysis. Phytohormone detection was performed by Shiseido SP HPLC-Thermo TSQ Quantum Ulta MS/Ms in sci-tech innovation company in Qingdao. All images are combined through Photoshop.

### Ethics statement

The tobacco varieties were supplied by Molecular Breeding Laboratory of Shihezi University (Tobacco, Xinjiang), including SR1 (*Nicotiana tabacum* cv. Petit Habana SR1) varieties. Experimental research and field studies on plants in this work, including the collection of plant material, comply with relevant institutional, national, and international guidelines and legislation.

## Supplementary Information

Supplementary Information 1.

Supplementary Information 2.

## Data Availability

The data and material that support the fndings of this study are available from the corresponding author on reasonable request.
