# Supplementary figures and images for: Transcriptome analysis provides insights into the molecular mechanism of GhSAMDC1 involving in rapid vegetative growth and early flowering in tobacco
Source: Sci Rep. 2022 Aug 10;12:13612. doi: 10.1038/s41598-022-18064-4 (PMC9365820; doi:10.1038/s41598-022-18064-4)

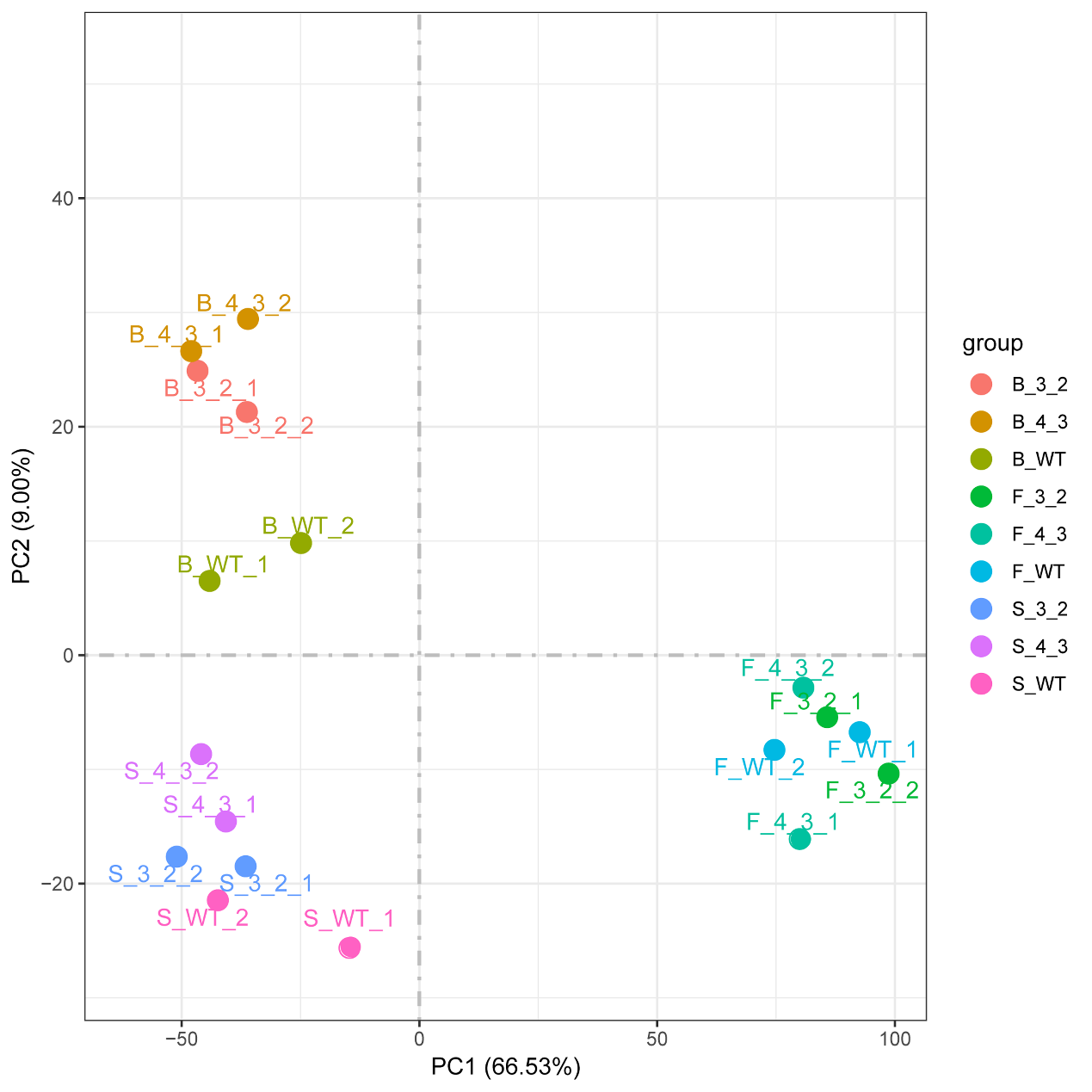


**Figure S1.** Principal Component Analysis (PCA) of the samples.

Supplement: Supplementary file 1 — Supplementary Information 1. [file 41598_2022_18064_MOESM1_ESM.docx]
