# Supplementary material for: Transcriptome analysis provides insights into the molecular mechanism of GhSAMDC1 involving in rapid vegetative growth and early flowering in tobacco
Source: Sci Rep. 2022 Aug 10;12:13612. doi: 10.1038/s41598-022-18064-4 (PMC9365820; doi:10.1038/s41598-022-18064-4)
Supplement: Supplementary file 2 — Supplementary Information 2. [file 41598_2022_18064_MOESM2_ESM.docx]

Table S1 Quality summary of sequencing data

| Sample name | Raw reads | Clean reads | clean bases | Error rate (%) | Q20(%) | Q30(%) | GC content (%) |
| --- | --- | --- | --- | --- | --- | --- | --- |
| S_WT_1 | 52385830 | 51214770 | 7.68G | 0.03 | 97.15 | 91.96 | 44.16 |
| S_WT_2 | 41888340 | 41033064 | 6.15G | 0.03 | 97.41 | 92.46 | 44.17 |
| S_3_2_1 | 55241844 | 54147878 | 8.12G | 0.03 | 97.31 | 92.26 | 43.99 |
| S_3_2_2 | 58462072 | 56763446 | 8.51G | 0.03 | 97.03 | 91.7 | 44.25 |
| S_4_3_1 | 51018574 | 48303544 | 7.25G | 0.03 | 97.36 | 92.43 | 44.15 |
| S_4_3_2 | 64023822 | 62615972 | 9.39G | 0.03 | 97.28 | 92.23 | 44.17 |
| B_WT_1 | 82339940 | 80829090 | 12.12G | 0.03 | 97.44 | 92.53 | 44.2 |
| B_WT_2 | 65881770 | 64182570 | 9.63G | 0.03 | 97.34 | 92.3 | 43.93 |
| B_3_2_1 | 53997308 | 51843482 | 7.78G | 0.03 | 97.25 | 92.2 | 44.08 |
| B_3_2_2 | 59766298 | 57904430 | 8.69G | 0.03 | 97.21 | 92.04 | 43.61 |
| B_4_3_1 | 51556670 | 50233344 | 7.54G | 0.03 | 97.06 | 91.77 | 43.66 |
| B_4_3_2 | 60394050 | 58711652 | 8.81G | 0.03 | 97.21 | 92.06 | 43.87 |
| F_WT_1 | 71710976 | 69879558 | 10.48G | 0.03 | 97.24 | 92.06 | 42.79 |
| F_WT_2 | 54439624 | 53347976 | 8.00G | 0.03 | 97.21 | 91.98 | 43.19 |
| F_3_2_1 | 59648224 | 58419114 | 8.76G | 0.03 | 97.2 | 91.95 | 42.92 |
| F_3_2_2 | 58539432 | 56761734 | 8.51G | 0.03 | 97.2 | 91.99 | 42.77 |
| F_4_3_1 | 59436726 | 58405904 | 8.76G | 0.03 | 97.26 | 92.15 | 42.97 |
| F_4_3_2 | 67745560 | 66258536 | 9.94G | 0.03 | 97.19 | 91.95 | 42.71 |

Table S2 FPKM distribution of different replicates and stages in wild type and transgenic lines

| FPKM Interval | 0~1 | 1~3 | 3~15 | 15~60 | >60 |
| --- | --- | --- | --- | --- | --- |
| S_WT_1 | 50480 (64.31%) | 12229 (15.58%) | 11357 (14.47%) | 3144 (4.01%) | 1286 (1.64%) |
| S_WT_2 | 43092 (54.90%) | 12393 (15.79%) | 16773 (21.37%) | 4570 (5.82%) | 1668 (2.12%) |
| S_3_2_1 | 42992 (54.77%) | 12459 (15.87%) | 16922 (21.56%) | 4582 (5.84%) | 1541 (1.96%) |
| S_3_2_2 | 44677 (56.92%) | 12471 (15.89%) | 15529 (19.78%) | 4197 (5.35%) | 1622 (2.07%) |
| S_4_3_1 | 44426 (56.60%) | 12453 (15.86%) | 15858 (20.20%) | 4279 (5.45%) | 1480 (1.89%) |
| S_4_3_2 | 44568 (56.78%) | 12566 (16.01%) | 15547 (19.81%) | 4185 (5.33%) | 1630 (2.08%) |
| B_WT_1 | 45426 (57.87%) | 12822 (16.33%) | 14583 (18.58%) | 4075 (5.19%) | 1590 (2.03%) |
| B_WT_2 | 42928 (54.69%) | 11981 (15.26%) | 16886 (21.51%) | 4987 (6.35%) | 1714 (2.18%) |
| B_3_2_1 | 46193 (58.85%) | 12752 (16.25%) | 14079 (17.94%) | 3908 (4.98%) | 1564 (1.99%) |
| B_3_2_2 | 41101 (52.36%) | 12302 (15.67%) | 18180 (23.16%) | 5125 (6.53%) | 1788 (2.28%) |
| B_4_3_1 | 45684 (58.20%) | 12751 (16.24%) | 14559 (18.55%) | 4017 (5.12%) | 1485 (1.89%) |
| B_4_3_2 | 42138 (53.68%) | 12561 (16.00%) | 17336 (22.09%) | 4876 (6.21%) | 1585 (2.02%) |
| F_WT_1 | 38973 (49.65%) | 10026 (12.77%) | 20530 (26.15%) | 7248 (9.23%) | 1719 (2.19%) |
| F_WT_2 | 40431 (51.51%) | 10709 (13.64%) | 19551 (24.91%) | 6192 (7.89%) | 1613 (2.05%) |
| F_3_2_1 | 38578 (49.15%) | 10499 (13.38%) | 20696 (26.37%) | 7069 (9.01%) | 1654 (2.11%) |
| F_3_2_2 | 38508 (49.06%) | 9486 (12.08%) | 20628 (26.28%) | 8144 (10.38%) | 1730 (2.20%) |
| F_4_3_1 | 41040 (52.28%) | 10625 (13.54%) | 18222 (23.21%) | 6682 (8.51%) | 1927 (2.45%) |
| F_4_3_2 | 39104 (49.82%) | 10249 (13.06%) | 20197 (25.73%) | 7209 (9.18%) | 1737 (2.21%) |

Table S3 Numbers of DEGs in transgenic line vs wild type at different stages

| Project | number of DEGs |
| --- | --- |
| S_3_2vsS_WT.DEG_down | 11 |
| S_3_2vsS_WT.DEG_up | 30 |
| S_4_3vsS_WT.DEG_down | 12 |
| S_4_3vsS_WT.DEG_up | 29 |
| B_3_2vsB_WT.DEG_down | 12 |
| B_3_2vsB_WT.DEG_up | 47 |
| B_4_3vsB_WT.DEG_down | 36 |
| B_4_3vsB_WT.DEG_up | 146 |
| F_3_2vsF_WT.DEG_down | 27 |
| F_3_2vsF_WT.DEG_up | 54 |
| F_4_3vsF_WT.DEG_down | 271 |
| F_4_3vsF_WT.DEG_up | 263 |
| total | 938 |

Table S4 GO annotation of the DEGs between 4-3 and WT at bolting stage

| GO accession | Description | Term type | Over represented *P _value_* | Corrected *P _value_* | DEG item | DEG list | Bg item | Bg list | Up | Down |
| --- | --- | --- | --- | --- | --- | --- | --- | --- | --- | --- |
| GO:0001071 | nucleic acid binding transcription factor activity | molecular function | 1.2696E-11 | 3.0794E-08 | 24 | 119 | 1841 | 49166 | 22 | 2 |
| GO:0003700 | transcription factor activity, sequence-specific DNA binding | molecular function | 1.2696E-11 | 3.0794E-08 | 24 | 119 | 1841 | 49166 | 22 | 2 |
| GO:0050794 | regulation of cellular process | biological process | 1.3733E-09 | 2.2206E-06 | 45 | 119 | 7411 | 49166 | 40 | 5 |
| GO:0050789 | regulation of biological process | biological process | 4.8449E-09 | 3.9695E-06 | 45 | 119 | 7699 | 49166 | 40 | 5 |
| GO:0080090 | regulation of primary metabolic process | biological process | 8.1349E-09 | 3.9695E-06 | 33 | 119 | 4533 | 49166 | 30 | 3 |
| GO:0006355 | regulation of transcription, DNA-templated | biological process | 1.0163E-08 | 3.9695E-06 | 31 | 119 | 4100 | 49166 | 28 | 3 |
| GO:1903506 | regulation of nucleic acid-templated transcription | biological process | 1.0163E-08 | 3.9695E-06 | 31 | 119 | 4100 | 49166 | 28 | 3 |
| GO:2001141 | regulation of RNA biosynthetic process | biological process | 1.0664E-08 | 3.9695E-06 | 31 | 119 | 4108 | 49166 | 28 | 3 |
| GO:2000112 | regulation of cellular macromolecule biosynthetic process | biological process | 1.1065E-08 | 3.9695E-06 | 32 | 119 | 4344 | 49166 | 29 | 3 |
| GO:0060255 | regulation of macromolecule metabolic process | biological process | 1.113E-08 | 3.9695E-06 | 33 | 119 | 4587 | 49166 | 30 | 3 |
| GO:0051252 | regulation of RNA metabolic process | biological process | 1.1515E-08 | 3.9695E-06 | 31 | 119 | 4123 | 49166 | 28 | 3 |
| GO:0010556 | regulation of macromolecule biosynthetic process | biological process | 1.1589E-08 | 3.9695E-06 | 32 | 119 | 4352 | 49166 | 29 | 3 |
| GO:0031326 | regulation of cellular biosynthetic process | biological process | 1.1589E-08 | 3.9695E-06 | 32 | 119 | 4352 | 49166 | 29 | 3 |
| GO:0009889 | regulation of biosynthetic process | biological process | 1.1985E-08 | 3.9695E-06 | 32 | 119 | 4358 | 49166 | 29 | 3 |
| GO:0065007 | biological regulation | biological process | 1.2274E-08 | 3.9695E-06 | 46 | 119 | 8226 | 49166 | 40 | 6 |
| GO:0051171 | regulation of nitrogen compound metabolic process | biological process | 1.3804E-08 | 4.1852E-06 | 32 | 119 | 4385 | 49166 | 29 | 3 |
| GO:0019219 | regulation of nucleobase-containing compound metabolic process | biological process | 1.5262E-08 | 4.2668E-06 | 31 | 119 | 4173 | 49166 | 28 | 3 |
| GO:0031323 | regulation of cellular metabolic process | biological process | 1.5832E-08 | 4.2668E-06 | 33 | 119 | 4665 | 49166 | 30 | 3 |
| GO:0010468 | regulation of gene expression | biological process | 1.7113E-08 | 4.3691E-06 | 32 | 119 | 4420 | 49166 | 29 | 3 |
| GO:0019222 | regulation of metabolic process | biological process | 4.1579E-08 | 0.000010085 | 33 | 119 | 4850 | 49166 | 30 | 3 |
| GO:0006351 | transcription, DNA-templated | biological process | 5.754E-08 | 0.000012688 | 33 | 119 | 4972 | 49166 | 30 | 3 |
| GO:0097659 | nucleic acid-templated transcription | biological process | 5.754E-08 | 0.000012688 | 33 | 119 | 4972 | 49166 | 30 | 3 |
| GO:0004512 | inositol-3-phosphate synthase activity | molecular function | 7.7372E-08 | 0.000014577 | 3 | 119 | 4 | 49166 | 0 | 3 |
| GO:0006021 | inositol biosynthetic process | biological process | 7.7372E-08 | 0.000014577 | 3 | 119 | 4 | 49166 | 0 | 3 |
| Continued Table S4 | | | | | | | | | | |
| GO:0046173 | polyol biosynthetic process | biological process | 7.7372E-08 | 0.000014577 | 3 | 119 | 4 | 49166 | 0 | 3 |
| GO:0032774 | RNA biosynthetic process | biological process | 7.813E-08 | 0.000014577 | 33 | 119 | 5034 | 49166 | 30 | 3 |
| GO:0034654 | nucleobase-containing compound biosynthetic process | biological process | 1.9757E-07 | 0.000035497 | 34 | 119 | 5516 | 49166 | 31 | 3 |
| GO:1901362 | organic cyclic compound biosynthetic process | biological process | 6.2655E-07 | 0.00010855 | 36 | 119 | 6343 | 49166 | 33 | 3 |
| GO:0046165 | alcohol biosynthetic process | biological process | 6.6454E-07 | 0.00011116 | 3 | 119 | 7 | 49166 | 0 | 3 |
| GO:0044271 | cellular nitrogen compound biosynthetic process | biological process | 8.6663E-07 | 0.00014013 | 40 | 119 | 7741 | 49166 | 34 | 6 |
| GO:0019438 | aromatic compound biosynthetic process | biological process | 1.2203E-06 | 0.00019096 | 34 | 119 | 5957 | 49166 | 31 | 3 |
| GO:0005578 | proteinaceous extracellular matrix | cellular component | 1.3645E-06 | 0.00020684 | 7 | 119 | 230 | 49166 | 7 | 0 |
| GO:0034645 | cellular macromolecule biosynthetic process | biological process | 1.4965E-06 | 0.00021999 | 40 | 119 | 7875 | 49166 | 34 | 6 |
| GO:0018130 | heterocycle biosynthetic process | biological process | 1.7836E-06 | 0.00025448 | 34 | 119 | 6053 | 49166 | 31 | 3 |
| GO:0031012 | extracellular matrix | cellular component | 1.8435E-06 | 0.00025551 | 7 | 119 | 241 | 49166 | 7 | 0 |
| GO:0009059 | macromolecule biosynthetic process | biological process | 0.000001987 | 0.00026775 | 40 | 119 | 7961 | 49166 | 34 | 6 |
| GO:0010467 | gene expression | biological process | 0.000003623 | 0.000475 | 40 | 119 | 8140 | 49166 | 35 | 5 |
| GO:0006020 | inositol metabolic process | biological process | 4.3448E-06 | 0.00055465 | 3 | 119 | 13 | 49166 | 0 | 3 |
| GO:0016070 | RNA metabolic process | biological process | 7.6971E-06 | 0.00095739 | 35 | 119 | 6716 | 49166 | 32 | 3 |
| GO:0044249 | cellular biosynthetic process | biological process | 0.000013781 | 0.0016629 | 44 | 119 | 9858 | 49166 | 36 | 8 |
| GO:1901576 | organic substance biosynthetic process | biological process | 0.000014054 | 0.0016629 | 45 | 119 | 10191 | 49166 | 37 | 8 |
| GO:0009058 | biosynthetic process | biological process | 0.000019165 | 0.0022136 | 46 | 119 | 10628 | 49166 | 38 | 8 |
| GO:0016872 | intramolecular lyase activity | molecular function | 0.000025813 | 0.0029121 | 3 | 119 | 23 | 49166 | 0 | 3 |
| GO:0044421 | extracellular region part | cellular component | 0.000030894 | 0.0034061 | 8 | 119 | 503 | 49166 | 8 | 0 |
| GO:0044262 | cellular carbohydrate metabolic process | biological process | 0.000049579 | 0.0053447 | 7 | 119 | 373 | 49166 | 2 | 5 |
| GO:1901617 | organic hydroxy compound biosynthetic process | biological process | 0.000077501 | 0.008173 | 3 | 119 | 33 | 49166 | 0 | 3 |
| GO:0019751 | polyol metabolic process | biological process | 0.00010713 | 0.011057 | 3 | 119 | 36 | 49166 | 0 | 3 |
| GO:0034637 | cellular carbohydrate biosynthetic process | biological process | 0.00033745 | 0.034104 | 5 | 119 | 230 | 49166 | 1 | 4 |
| GO:0005576 | extracellular region | cellular component | 0.00046163 | 0.045701 | 13 | 119 | 1828 | 49166 | 11 | 2 |

Table S5 GO annotation of the DEGs between 4-3 and WT at flowering stage

| GO accession | Description | Term type | Over represented *P _value_* | Corrected *P _value_* | DEG item | DEG list | Bg item | Bg list | Up | Down |
| --- | --- | --- | --- | --- | --- | --- | --- | --- | --- | --- |
| GO:0010333 | terpene synthase activity | molecular function | 5.35E-11 | 2.60E-07 | 13 | 354 | 148 | 49166 | 13 | 0 |
| GO:0016838 | carbon-oxygen lyase activity, acting on phosphates | molecular function | 1.32E-10 | 3.19E-07 | 13 | 354 | 158 | 49166 | 13 | 0 |
| GO:0019295 | coenzyme M biosynthetic process | biological process | 3.02E-07 | 0.000304 | 3 | 354 | 3 | 49166 | 0 | 3 |
| GO:0019296 | coenzyme M metabolic process | biological process | 3.02E-07 | 0.000304 | 3 | 354 | 3 | 49166 | 0 | 3 |
| GO:0016835 | carbon-oxygen lyase activity | molecular function | 3.13E-07 | 0.000304 | 15 | 354 | 406 | 49166 | 15 | 0 |
| GO:0000287 | magnesium ion binding | molecular function | 2.80E-06 | 0.002268 | 13 | 354 | 353 | 49166 | 13 | 0 |
| GO:0000902 | cell morphogenesis | biological process | 3.82E-06 | 0.002315 | 9 | 354 | 156 | 49166 | 0 | 9 |
| GO:0032989 | cellular component morphogenesis | biological process | 3.82E-06 | 0.002315 | 9 | 354 | 156 | 49166 | 0 | 9 |
| GO:0048869 | cellular developmental process | biological process | 1.10E-05 | 0.005918 | 12 | 354 | 331 | 49166 | 1 | 11 |
| GO:0006457 | protein folding | biological process | 2.62E-05 | 0.012087 | 11 | 354 | 330 | 49166 | 0 | 11 |
| GO:0016829 | lyase activity | molecular function | 2.74E-05 | 0.012087 | 21 | 354 | 1022 | 49166 | 20 | 1 |
| GO:0055114 | oxidation-reduction process | biological process | 3.28E-05 | 0.013277 | 57 | 354 | 4485 | 49166 | 43 | 14 |
| GO:0016491 | oxidoreductase activity | molecular function | 3.80E-05 | 0.014178 | 59 | 354 | 4746 | 49166 | 45 | 14 |
| GO:0051082 | unfolded protein binding | molecular function | 4.16E-05 | 0.014403 | 11 | 354 | 344 | 49166 | 1 | 10 |
| GO:0009653 | anatomical structure morphogenesis | biological process | 6.73E-05 | 0.021771 | 10 | 354 | 288 | 49166 | 0 | 10 |

Table S6 KEGG pathways of the DEGs in wild-type and transgenic

| Term | Database | ID | Input number | Background number | *P _value_* | Corrected *P _value_* | KEGG ID/KO |
| --- | --- | --- | --- | --- | --- | --- | --- |
| B_3_2vsB_WT | | | | | | | |
| Histidine metabolism | KEGG PATHWAY | sly00340 | 1 | 31 | 0.01176 | 0.047039 | sly:101244621\| |
| B_4_3vsB_WT | | | | | | | |
| Plant-pathogen interaction | KEGG PATHWAY | sly04626 | 6 | 176 | 0.00024 | 0.006009 | sly:101259138\|sly:101256237\|sly:101259138\|sly:100316879\|sly:101255501\|sly:101256237\| |
| F_4_3vsF_WT | | | | | | | |
| Protein processing in endoplasmic reticulum | KEGG PATHWAY | sly04141 | 58 | 238 | 9.90E-38 | 6.04E-36 | sly:101268307\|sly:101260143\|sly:101254946\|sly:544282\|sly:101253115\|sly:101254866\|sly:101268271\|sly:543846\|sly:101266349\|sly:543507\|sly:101255164\|sly:544282\|sly:544282\|sly:101268307\|sly:543848\|sly:544024\|sly:101255164\|sly:101266525\|sly:101265819\|sly:544282\|sly:544282\|sly:101055596\|sly:544282\|sly:544282\|sly:544282\|sly:101264183\|sly:101264936\|sly:101252822\|sly:101268307\|sly:101266525\|sly:101256272\|sly:101268307\|sly:543507\|sly:544282\|sly:101255185\|sly:544205\|sly:101253115\|sly:101268271\|sly:101259708\|sly:543902\|sly:101265819\|sly:101261304\|sly:101264183\|sly:544282\|sly:101256536\|sly:543902\|sly:101268307\|sly:544282\|sly:101264183\|sly:543848\|sly:101055596\|sly:101266525\|sly:544205\|sly:544282\|sly:544282\|sly:101268307\|sly:101248279\|sly:544282\| |
| alpha-Linolenic acid metabolism | KEGG PATHWAY | sly00592 | 10 | 49 | 9.61E-07 | 2.93E-05 | sly:100301936\|sly:100736436\|sly:544239\|sly:101261255\|sly:101251665\|sly:100301936\|sly:101261255\|sly:101244897\|sly:543642\|sly:544008\| |
| Spliceosome | KEGG PATHWAY | sly03040 | 17 | 185 | 5.21E-06 | 0.000106 | sly:101257217\|sly:101255164\|sly:101257012\|sly:101245917\|sly:101055596\|sly:101055596\|sly:101254866\|sly:101263748\|sly:101245917\|sly:101263601\|sly:101267415\|sly:101245917\|sly:101245917\|sly:101255185\|sly:101263601\|sly:101255164\|sly:101266050\| |
| Sesquiterpenoid and triterpenoid biosynthesis | KEGG PATHWAY | sly00909 | 4 | 15 | 0.000866 | 0.013199 | sly:101251277\|sly:101257190\|sly:101264676\|sly:101251277\| |
| Linoleic acid metabolism | KEGG PATHWAY | sly00591 | 4 | 18 | 0.001546 | 0.018857 | sly:100301936\|sly:100301936\|sly:101244897\|sly:544008\| |

Table S7 Relative expression level of the 6 selected genes at the three stages determined by RNA-Seq analysis

| Gene ID | S_WT | S_3_2 | S_4_3 | B_WT | B_3_2 | B_4_3 | F_WT | F_3_2 | F_4_3 |
| --- | --- | --- | --- | --- | --- | --- | --- | --- | --- |
| LOC107786068 | 0.000 | 1.592 | 1.399 | 0.000 | 1.455 | 1.201 | 0.000 | 4.191 | 2.717 |
| LOC107778275 | 0.009 | 3.783 | 6.198 | 0.000 | 1.571 | 2.179 | 0.005 | 2.660 | 2.514 |
| LOC107784270 | 0.000 | 7.447 | 5.264 | 0.259 | 4.849 | 3.361 | 0.101 | 14.302 | 10.395 |
| LOC107781406 | 1.580 | 0.000 | 0.000 | 1.983 | 0.217 | 0.214 | 6.825 | 0.955 | 1.850 |
| LOC107821752 | 5.641 | 0.005 | 0.000 | 7.586 | 0.005 | 0.025 | 15.283 | 0.599 | 0.029 |
| LOC107793800 | 2.014 | 0.066 | 0.000 | 2.306 | 0.000 | 0.013 | 18.419 | 1.943 | 2.183 |

Table S8 Summary of transcription factor genes in DEGs

| No | Family | number of transcription factor genes |
| --- | --- | --- |
| 1 | AP2-EREBP | 14 |
| 2 | WRKY | 9 |
| 3 | HSF | 6 |
| 4 | Tify | 6 |
| 5 | GRAS | 4 |
| 6 | MYB | 2 |
| 7 | NAC | 2 |
| 8 | TAZ | 2 |
| 9 | MBF1 | 2 |
| 10 | bHLH | 1 |
| 11 | bZIP | 1 |
| 12 | mTERF | 1 |
| 13 | GARP-G2-like | 1 |
| 14 | PHD | 1 |
| 15 | SBP | 1 |
| 16 | C2C2-CO-like | 1 |
| 17 | HMG | 1 |
| 18 | Pseudo ARR-B | 1 |

Table S9 Sample information of plant materials for transcriptome analysis

| Sample name | Name of plant materials |
| --- | --- |
| WT seedling (30 d) | S_WT |
| 3-2 seedling (30 d) | S_3_2 |
| 4-3 seedling (30 d) | S_4_3 |
| WT bolting (60 d) | B_WT |
| 3-2 bolting (60 d) | B_3_2 |
| 4-3 bolting (60 d) | B_4_3 |
| WT flowering (90 d) | F_WT |
| 3-2 flowering (90 d) | F_3_2 |
| 4-3 flowering (90 d) | F_4_3 |

Table S10 qRT-PCR primers list

| Gene id | Sense primer | Antisense primer |
| --- | --- | --- |
| LOC107778275 | GATAGCAGACCTTGACGCGA | TTGTCGGGATGTAGGCTTGC |
| LOC107784270 | GGTATCGACAACAACAGGAGGA | CGCTTATATGCCTGACATCATCC |
| LOC107786068 | GTCACACTGCAGGACATGGA | CGCGCGTATGTACTTGTTGG |
| LOC107781406 | CAGTCGACGGTCCTCAACAA | CCAAAATTGGGTCCAGCTGC |
| LOC107821752 | CGATCTCGGGCATGGGATTT | CCAATGGGTTGTGATGCACG |
| LOC107793800 | CGATTCTCCAATTCAGCCATTCC | TTAGCAGCCCCATTGGTTGT |
| *Actin* (X69885) | CCTGAGGTCCTTTTCCAACCA | GGATTCCGGCAGCTTCCATT |
